# Bridging AMR knowledge gaps and improving policy implementation: a perspective on the role of community engagement in Africa

**DOI:** 10.3389/fpubh.2025.1668830

**Published:** 2025-11-21

**Authors:** Kartik Mihir Mishra, A. Tracie Muraya, Niniola Williams, Tapiwanashe Kujinga, Raphael Chanda, Estelle Mbadiwe, Hafeez Hamza, Kayla M. Strong, Geneviève Boily-Larouche, Arne Ruckert

**Affiliations:** 1 AMR Policy Accelerator, Global Strategy Lab, York University, Toronto, ON, Canada; 2 ReAct Africa, Nairobi, Kenya; 3 Dr. Ameyo Stella Adadevoh (DRASA) Health Trust, Abuja, Nigeria; 4 Pan-African Treatment Access Movement, Harare, Zimbabwe; 5 ReAct Africa, Lusaka, Zambia; 6 Ducit Blue Foundation, Department of Research and Analysis, Abuja, Nigeria; 7 Ducit Blue Foundation, Department of Research and Analysis, Nairobi, Kenya

**Keywords:** antimicrobial resistance, community engagement and one health in Africa, citizen science, equity, implementation, partnerships

## Abstract

Antimicrobial resistance (AMR) is an escalating global threat, posing a serious challenge to public health and medical and social advancements. Top-down research methods and policy implementation approaches have fallen short in capturing the complexities of AMR transmission and supporting effective policy implementation, including in diverse and resource-constrained settings. This perspective article suggests that community engagement has the potential to improve AMR outcomes by generating more community-relevant, context-specific AMR knowledge, providing novel data and evidence for action. We further note that locally rooted civil society organizations (CSOs) are essential to fostering relevance, acceptability, and effectiveness of AMR interventions, as well as engendering strong commitment to AMR policy development and implementation. We focus on the role that lived experiences and participatory research approaches, such as citizen science, can play in generating locally grounded AMR knowledge, as well as how community engagement can facilitate trust and a sense of ownership surrounding AMR policies among community members. We also note the potential of community engagement to identify and address equity and cross-sectoral coordination challenges and contribute to more equitable and sustainable AMR policy implementation. Drawing on successful initiatives in Nigeria, Zimbabwe, Malawi, and Kenya, and acknowledging the role of advocacy by CSOs, we demonstrate the potential of community-driven approaches to transform AMR responses and human, animal, and environmental health-related outcomes, and note that to be successful, this community engagement must be genuine, meaningful, inclusive, and transparent.

## Introduction

Antimicrobial resistance (AMR) is one of the most pressing global health challenges of our time, with more than 1 million deaths annually directly attributable to bacterial AMR (1). While the factors influencing the development and transmission of resistance are not fully understood globally, socio-structural drivers embedded in communities, such as poverty, health system inequities, and challenging regulatory environments, play a critical role in shaping antimicrobial use and resistance patterns (2). In this context, community engagement is an essential strategy for addressing the root causes of AMR and improving the reach, relevance, acceptability, effectiveness, and implementation of AMR policies (3–5). AMR mitigation requires a whole-of-society approach, which includes the engagement of diverse community members. We define community in the context of AMR as a diverse group of people—individuals, families, local leaders, civil society organizations, and institutions—who are directly affected by, or engaged in, behaviors, norms, and systems that influence antimicrobial use and resistance within specific social, cultural, and geographic contexts. AMR mitigation efforts at global and national levels have been predominantly top-down, resulting in poorly tailored interventions and implementation challenges, highlighting the need for stronger community engagement in policy development (5).

In this perspective article, we suggest that community engagement can improve AMR interventions and human, animal and environmental health outcomes by generating a deeper understanding of the unique and complex community drivers of AMR, which in turn can lead to the development of more relevant and acceptable AMR policies and effective implementation of AMR interventions in community settings. We further note that fostering strong stakeholder commitments for AMR policy development and implementation in the community requires meaningful engagement with locally rooted civil society organizations (CSOs). To support this argument, we first discuss how integrating lived experiences, participatory research, and citizen science into AMR research and knowledge production can foster more locally rooted knowledge connected directly to the community. We then describe how community engagement can raise awareness of multi-faceted AMR challenges, as well as engender a sense of trust and policy ownership within the community, which in turn can help create stronger commitments to develop and implement proposed AMR interventions. We finally discuss the role of community engagement in identifying and addressing equity and multi-sectoral coordination challenges, and the potential of this approach to lead to more equitable, sustainable, and One Health informed AMR policy implementation practices. We draw on experiences from civil society organizations on the ground, including the Pan African Treatment Access Movement (PATAM), DRASA Health Trust, Ducit Blue Foundation, and ReAct Africa, to support our argument. These organizations have been at the forefront of advocating for community-led solutions to AMR in Sub-Saharan Africa (SSA), recognizing the importance of involving those most affected in research, policy development and implementation. By engaging with communities directly, CSOs can draw on and cater to local knowledge and lived experiences to inform culturally and contextually relevant AMR interventions, enhance the relevance, acceptability and effectiveness of AMR strategies and foster more sustainable AMR policy solutions (4).

Taken together, this perspective article thus advances three interlinked arguments: that community engagement can enhance AMR policy design by (1) integrating lived experiences and local knowledge into AMR research and policymaking, (2) strengthening trust, awareness, and shared ownership of interventions, and (3) promoting equity and multisectoral collaboration aligned with One Health principles. We use examples from civil society organizations in Sub-Saharan Africa, based on the personal experiences of some of the co-authors, to illustrate how these dimensions collectively support more contextually grounded and sustainable AMR responses.

## Harnessing lived experiences to improve AMR interventions

A particularly promising approach within community engagement is the integration of more participatory research approaches, including citizen science, which empower local communities to actively contribute to the research process by designing the methodology, collecting, analyzing, and interpreting evidence. Citizen science can not only democratize research but can also ensure that AMR evidence is rooted in local realities, making it more relevant for shaping policies and interventions (6).

In Malawi, the ICARS Responsive Dialog Project drew on the knowledge of local stakeholders, including healthcare professionals and community leaders, to generate evidence that informed both policy decisions and community-led AMR solutions (7). The initiative highlighted the need for community buy-in and stressed that interventions are most effective when they are co-created with those who experience the problem firsthand. For instance, through focus group discussions and participatory learning exercises, stakeholders provided crucial insights into antibiotic misuse and the social behaviors that drive resistance (7). In Zambia, similar efforts were made as community members actively contributed to the improvement of antibiotic use and surveillance through Participatory Learning and Action (PLA) techniques (7, 8). PLA involves communities in problem identification, solution generation, and the monitoring of intervention outcomes, creating a feedback loop between the affected population and the implementers (7). The project in Zambia revealed complex social dynamics, such as the influence of gender roles on antibiotic access and usage, which were crucial for understanding AMR transmission in the region (7). By gathering data from these communities, the project was able to generate evidence to tailor interventions to address these nuanced, context-specific factors, making AMR strategies more effective, an approach that has been proven successful in the past for other communicable diseases (3).

In Kenya, the Africa Voices Foundation (AVF) *Citizen-Generated Data to Address AMR in Kenya* project engaged 5,313 community members across Bungoma, Kiambu, and Kilifi counties to gather local views on the drivers of inappropriate antibiotic use (8). The data is generated through live interactive radio shows, key informant interviews, focus group discussions and interactive short-message-services (SMS) surveys. From this AMR-focused project, the Citizen-Generated Data (CGD) highlighted the crucial role of effective community engagement in not only bringing to the fore the nuances associated with hard-to-change behaviors but also providing platforms for citizen-government engagement (9). Since 2019, ReAct Africa, in collaboration with Ace Africa, has further engaged elementary level school children in Busia, Kisumu and Busia counties through the child-to-child learning methodology, art, skits, and sports programs, and by establishing kitchen gardens (9). These programs have resulted in locally-based and culturally accepted solutions to inappropriate use of antibiotics among the children and communities associated with the project school, leading to advocacy to include child-appropriate modules in the primary school curriculum by education stakeholders such as the project schools’ administration, teachers, Ministry of Education and Ministry of Public Health representatives in the project counties (9). Similarly, ReAct Africa’s engagement with university students and young graduates through training workshops and webinars has resulted in context-based AMR solutions, influencing regional AMR policies through Africa youth-developed AMR policy papers and the establishment of youth-led institutions such as AMR Now Initiative in Kenya (10). These examples from Kenya, Malawi and Zambia demonstrate the transformative potential of involving communities in mitigating AMR (10). By embedding participatory research approaches in AMR interventions, the projects have successfully uncovered previously overlooked drivers of resistance, enabling a more targeted and effective approach to combating the problem.

However, while citizen-generated data offers valuable local insights, it can be affected by reporting bias and variable quality. Additionally, ensuring standardized collection methods and validation is essential to maintain reliability. For instance, ethical considerations, such as informed consent, data security, and managing community expectations must help guide engagement to avoid tokenism. Integrating community-derived evidence into formal AMR surveillance frameworks like GLASS, can help bridge local participation with institutional monitoring, which would strengthen both inclusivity and data integrity.

## Raising community awareness for AMR

Raising awareness about AMR is an essential component of AMR community engagement strategies, particularly when AMR interventions rely on community support for their successful implementation. In this context, community-led initiatives that raise awareness, localize messaging, and empower citizens to participate in AMR prevention and mitigation efforts can significantly improve the reach, relevance, acceptability, and sustainability of AMR policies.

In Nigeria, the Ducit Blue Foundation and Dr. Ameyo Stella Adadevoh (DRASA) Health Trust have been instrumental in advocating for community-centered AMR awareness interventions, which have contributed to improved antibiotic stewardship, local capacity-building and behavior change. For example, DRASA’s youth engagement program, which has established Health and Hygiene Clubs in secondary schools in two states, develops students into AMR Ambassadors who are responsible for increasing awareness and promoting preventive behaviors within their circles of influence, including schools, homes, and neighborhoods (11). Their ownership is demonstrated by the most recent cohort of Ambassadors who conducted independent activities outside the Club to influence the health practices of those around them, with each Ambassador reaching an average of nine other people – demonstrating the multiplier effect of equipping community members as AMR change agents (12, 13). These initiatives have helped foster greater ownership of AMR interventions at the community level, leading to more sustainable outcomes (12). Similarly, the Ducit Blue Foundation (DBF), the NGO arm of Ducit Blue Solutions, empowers youth to bridge AMR knowledge gaps in African communities through their award-winning internship/mentorship program (14). In collaboration with Triple-A, the Foundation recently engaged 30 youth from diverse African countries in capacity-building sessions focused on communicating AMR and community engagement, resulting in the creation of infographics with actionable infection prevention steps (15). These were translated into over 50 African languages and disseminated in local markets, farms, schools, and among religious groups during World Antimicrobial Awareness Week (WAAW), extending AMR awareness to grassroots levels (13). Volunteers reported practical challenges such as antibiotic misuse and reliance on unregulated medicine, underscoring the need for targeted AMR education interventions (12). Leveraging the mantra “Know, Stop, Save the Future,” DBF also engaged students and teachers in debates and handwashing demonstrations to enable community-led AMR solutions and antibiotic stewardship. These discussions brought to light practical challenges, including reports of antibiotics being found in children’s school bags, which were intended for siblings or parents, and a widespread preference for unregulated medicines due to their affordability (16). A key conclusion from the dialog was the urgent need to enforce antibiotic sales regulations as a critical step toward curbing misuse and safeguarding public health (12). The initiative concluded with students pledging to be antibiotic ambassadors in their communities, with awards presented to outstanding participants, an effort recognized at the Antibiotic Guardian Awards (16).

## Building trust and ownership: ramping up community engagement in NAPs

Community engagement is essential for the development and successful implementation of National Action Plans (NAPs) on AMR (5, 17). Without the active involvement of communities, efforts to improve awareness, change behaviors, and promote responsible antimicrobial use risk falling short. Recognizing this, several African countries have begun integrating community-driven approaches into their NAPs on AMR, working with local leaders, youth groups, faith-based organizations, and grassroots networks to strengthen public awareness and ownership of AMR interventions (5).

In October 2024, Nigeria launched its second-generation NAP 2.0 on AMR. In this plan, community engagement is integrated as one of the eight guiding principles that underpin the foundation and implementation of Nigeria’s NAP 2.0, with a focus on collaborating with youth groups, village elders’ forums, and faith-based organizations (18). The development of Nigeria’s second NAP included mapping and engagement of CSOs and community-level stakeholders and their subsequent inclusion in every phase of the NAP development process, to ensure a contextualized approach to AMR response across the country. The participation of these CSOs, who are traditionally overlooked in AMR policy development, ensured that Nigeria’s new NAP was designed with bottom-up input (18). Similarly, in Zimbabwe, the government has implemented a National AMR Strategy (2017–2022), with a focus on public education, awareness, and infection prevention and control interventions. The NAP emphasizes that for interventions to be successful, they require involvement of local communities, for example, through the development of AMR focal points at the district level (19). This strategy has helped build some ownership and accountability for the success of AMR interventions in Zimbabwe, but with limitations related to the lack of institutionalization of this approach through a standing One Health governance infrastructure (20).

## Box1. Community engagement and its alignment with AMR NAP objectives

1. Awareness and education

Community engagement can contribute to the development of local awareness about the need for AMR interventions and contextually resonant AMR messaging, as seen in the work of the DRASA Health Trust and Ducit Blue Foundation who engage schools and youth groups as AMR Ambassadors in Nigeria to raise AMR awareness.

2 Surveillance and reporting

Community engagement can strengthen surveillance through citizen-generated data (as seen in Kenya’s Africa Voices Foundation model) and could be adapted for other countries to enable bottom-up AMR/AMU reporting.

3 Infection prevention and control (IPC)

Community members can be trained as IPC champions in schools, markets, and farms, an approach embodied within DRASA’s Health and Hygiene Clubs in Nigerian schools and with Zimbabwe’s emphasis on district-level IPC interventions. These champions can reinforce hand hygiene, waste disposal, and safe antimicrobial handling, helping localize IPC practices and anchor them in community settings.

4 Antimicrobial stewardship

CSO-led community education and dialog can help identify and address behavioral and structural drivers of antibiotic misuse. This can be seen in the examples of Nigerian youth and community ambassadors which serve as stewardship advocates, and Zimbabwe’s district-level coordination mechanisms which have integrated similar participatory learnings to promote prudent antimicrobial use among human and animal health workers.

5 Research and innovation

Citizen science and participatory action research can be applied to uncover local AMR drivers and co-create AMR solutions. This process should integrate traditional knowledge systems and lived experience into AMR innovation and monitoring frameworks. Partnering with CSOs and academic institutions to implement participatory and citizen science models, similar to those described in Malawi and Zambia, can generate locally grounded evidence for AMR solutions.

6 Governance and financing

Community engagement and partnering with local CSOs can help institutionalize community representation and broker trust between governments and communities, improving governance and accountability and facilitating better resource coordination and allocation. Nigeria’s inclusion of CSOs and community stakeholders in all phases of NAP 2.0 design institutionalizes community voice, while Zimbabwe’s district focal points provide a governance interface for local accountability.

## Toward a holistic approach: integrating equity and one health

The integration of equity and the One Health approach, which recognizes the interconnectedness of human, animal, and environmental health, is critical to mitigating AMR, and both equity and One Health must lie at the center of community engagement strategies for AMR mitigation (7). By bringing together stakeholders from human health, veterinary medicine, and environmental sciences, the One Health approach creates a comprehensive strategy that tackles AMR from multiple angles. This integrated framework ensures that community-driven solutions are not only scientifically informed but also inclusive of all sectors impacted by AMR (7).

Equity-focused interventions in both Nigeria and Zimbabwe further demonstrate how prioritizing community engagement can lead to more inclusive and sustainable AMR solutions. In Nigeria, the national surveillance network for AMR and in Zimbabwe, district-level AMR focal points, have been specifically designed to include rural and underserved populations, which are frequently left out of top-down AMR strategies (18, 19). Additionally, to reach underserved populations, Nigeria aims to leverage existing community structures by conducting AMR training for Community Health Workers, Community Animal Health Workers, and Community Health Extension Workers, among others (18). The Pan African Treatment Access Movement’s (PATAM) and other CSO advocacy for marginalized populations and cross-sector collaboration complement such efforts, highlighting the potential for community-driven AMR solutions to address both scientific and social challenges (18). The inclusion of traditionally underrepresented voices, such as those most affected by AMR, can ensure that interventions are more equitable and contextually relevant. In this way, community engagement becomes a vital mechanism for fostering trust, promoting shared ownership of AMR interventions, and creating lasting solutions across diverse sectors (4).

## Conclusion

Our examples of community engagement demonstrate the promise and complexity of community engagement in AMR mitigation. Across contexts, engagement with community members can help better define the drivers of AMR, raise awareness about AMR in community settings, and develop and implement contextually appropriate AMR solutions. Community engagement is not new to public health or AMR, but its systematic integration into AMR policymaking and implementation remains limited, with some progress documented in this article. Drawing on these experiences, future AMR initiatives should organize community engagement through organic partnerships with locally rooted CSOs, use participatory and citizen science methods tailored to diverse groups, including youth, women, and others with heightened AMR vulnerabilities, and integrate continuous feedback mechanisms to build trust and accountability.

When done poorly, community engagement risks tokenism and may erode trust and create community fatigue, especially if expectations are mismanaged or voices are not genuinely heard. The examples reviewed illustrate that successful community engagement requires sustained trust-based relationships, mutual accountability, and an openness to discomforting insights that may challenge top-down assumptions. Locally rooted CSOs have shown capacity to lead this work effectively by acting as key actors in the process, bridging and brokering community realities to policy processes. Yet, ‘community’ is not monolithic as age, gender, livelihood, literacy, and social position all shape people’s priorities and engagement preferences. Future AMR research is needed to empirically evaluate community engagement approaches, including optimal participant composition, meaningful engagement processes, and contextual and situational barriers and facilitators, to inform scalable and equitable AMR interventions. Strategies should be explicit about which groups are engaged, through what methods, and to what end, with what specific outcomes. Recognizing these distinctions is essential to moving from *ad hoc* engagement to equitable, trusted, and context-sensitive AMR action that relies on community perspectives. Experience of community engagement for other communicable diseases, including HIV, can inform how the AMR community can improve its engagement process for more effective, relevant and acceptable AMR policymaking.

## Data Availability

The original contributions presented in the study are included in the article/supplementary material, further inquiries can be directed to the corresponding author/s.
